# Natural Products and Biological Activity of the Pharmacologically Active Cauliflower Mushroom *Sparassis crispa*

**DOI:** 10.1155/2013/982317

**Published:** 2013-03-18

**Authors:** Takashi Kimura

**Affiliations:** Research & Development Center, Unitika Ltd., 23 Uji-Kozakura, Uji, Kyoto 611-0021, Japan

## Abstract

*Sparassis crispa*, also known as cauliflower mushroom, is an edible mushroom with medicinal properties. Its cultivation became popular in Japan about 10 years ago, a phenomenon that has been attributed not only to the quality of its taste, but also to its potential for therapeutic applications. Herein, I present a comprehensive summary of the pharmacological activities and mechanisms of action of its bioactive components, such as beta-glucan, and other physiologically active substances. In particular, the immunomodulatory mechanisms of the beta-glucan components are presented herein in detail.

## 1. Introduction

Medicinal mushrooms have an established history of use in traditional Asian therapies. Over the past 2 to 3 decades, scientific and medical research in Japan, China, and Korea, and more recently in the United States, has increasingly demonstrated the potent and unique properties of compounds extracted from mushrooms for the prevention and treatment of cancer and other chronic diseases. Various important pharmaceutical products with proven medicinal applications have been derived from mushrooms [1].

**Figure 1:**
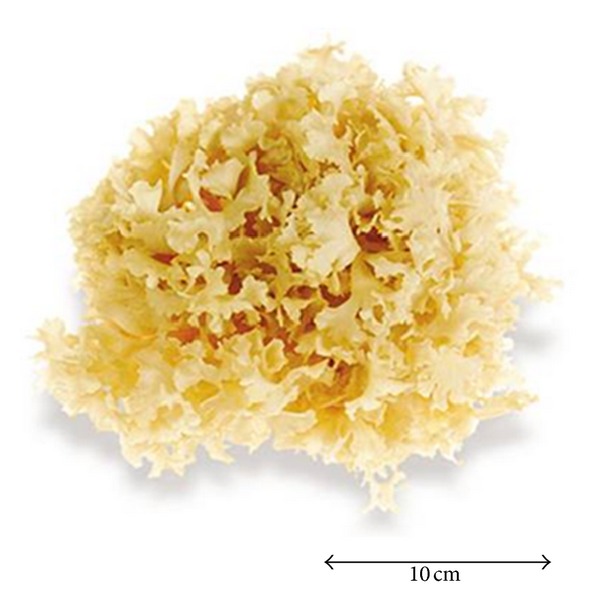
*Sparassis crispa* Wulf.:Fr. [2].

*Sparassis crispa *Wulf.:Fr. (Figure 1), also known as cauliflower mushroom, is an edible mushroom with various medicinal properties whose cultivation has recently become popular in Japan. The taxonomy of *S. crispa *is as follows: kingdom, Fungi; phylum, Basidiomycota; class, Agaricomycetes; order, Polyporales; family, Sparassidaceae; genus, *Sparassis*; and species, *crispa*. It is a brown-rot fungus that primarily grows on the stumps of coniferous trees and is widely distributed throughout the North Temperate Zone. *S. crispa* has been reported to have many biological activities, which are detailed below.

## 2. Chemical Constituents and Bioactive Components of *S. crispa*

Scientific investigation has led to the isolation of many compounds from *S. crispa* that have been shown to have health-promoting activities. The fruiting bodies of *S. crispa* contain approximately 90% water, protein, lipid, carbohydrate, ash, and dietary fiber (Table 1) [3]. Furthermore, the content of vitamin D_2_, which aids intestinal calcium absorption, was shown to be 0.17 mg per 100 g of dry weight, a concentration that is higher than that observed in other mushrooms [4]. Also *S. crispa* contained a relatively large amount of glucosyl ceramide (approximately 0.2%), which is a glycoside of ceramide. It was demonstrated that the moiety of sphingoid base was characterized by the unique structure [5]. Though *S. crispa* has a scent of its own, the results of headspace analyses showed that 3-octanone, DL-3-octanol, and 1-octen-3-ol contributed mutually to the particular aroma of this mushroom [6]. It is noteworthy that the beta-glucan content of *S. crispa* is more than 40% of the dry weight of the fruiting bodies, as measured by the enzyme method of the Japan Food Research Laboratories (Tokyo) [3].

### 2.1. Polysaccharide (Beta-Glucan)

#### 2.1.1. Primary Structure

**Figure 2:**
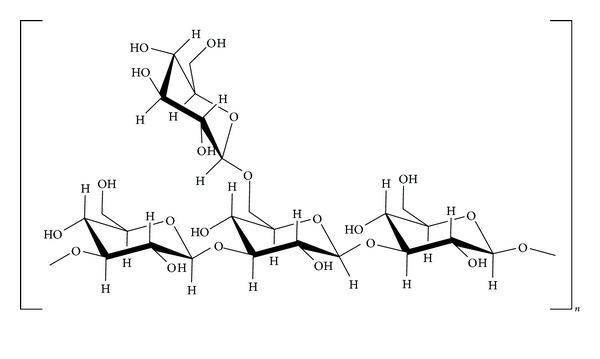
Chemical structure of SCG[9].

Using chemical, enzymatic, and NMR analyses, it was shown that the primary structure of a purified beta-glucan (designated SCG), obtained from cultured fruiting bodies of *S. crispa* is a 6-branched 1,3-beta-glucan, with one branch in approximately every 3 main chain units (Figure 2) [7–9].

#### 2.1.2. Biological Activities

Tumor size in cancerous (Sarcoma 180) ICR mice was dose-dependently decreased after 5 weeks of oral administration of *S. crispa* (10 or 100 mg/kg) in comparison to a control group. Furthermore, the survival rate of these model mice was higher when similarly treated with *S. crispa* [2]. Since SCG content in dry powder of *S. crispa* was measured to be more than 40%, SCG was likely be responsible for this antitumor effect.

Ohno et al. prepared polysaccharide fractions from the fruiting bodies of cultured *S. crispa* and showed their antitumor activity against the solid form of Sarcoma 180 in ICR mice with strong vascular dilation and hemorrhage reactions [7]. Furthermore, intraperitoneal and oral SCG over a wide range of concentrations enhanced hematopoietic responses in mice with leukopenia induced by cyclophosphamide (CY, a DNA-alkylating agent) [10, 11]. This effect was augmented in combination with isoflavone aglycone [12]. SCG was also shown to stimulate leukocytes to produce cytokines such as IL-8 in whole-cell cultures of human peripheral blood [13] and in mouse splenocytes [14].

Yamamoto et. al reported antiangiogenic and antimetastatic effects of SCG on neoplasm by using different animal models [8]. Oral administration of SCG suppressed B16-F10 cell-induced angiogenesis in a dorsal air sac assay using ICR mice and suppressed vascular endothelial growth factor induced neovascularization in a Matrigel plug assay using C57BL/6J mice. Furthermore, it suppressed the growth and number of metastatic tumor foci in the lung, along with primary tumor growth in a C57BL/6J mice model of spontaneous metastasis. From these findings, it is apparent that the oral administration of SCG exerts a suppressive effect on tumor growth and metastasis in the lung through the inhibition of tumor-induced angiogenesis.

Taken together, these results demonstrate that SCG exhibits various biological activities, including antitumor effects, enhancement of the hematopoietic response, and induction of cytokine production *in vivo* and *in vitro*.

#### 2.1.3. Mechanisms

Harada et al. reported strain-specific differences of the reactivity of mice to SCG, with DBA/1 and DBA/2 mice being highly sensitive to SCG. In splenocytes derived from various inbred strains of mice, interferon-*γ* (IFN-*γ*) production was not induced by SCG. However, splenocytes from naïve DBA/1 and DBA/2 mice strongly react with SCG to produce IFN-*γ* [14]. Furthermore, in addition to IFN-*γ*, cytokines induced by SCG were screened for and found to include tumor necrosis factor-*α* (TNF-*α*), granulocyte-macrophage colony-stimulating factor (GM-CSF), and interleukin-12 (IL-12p70) [15]. Since the sera of naïve DBA/1 and DBA/2 mice contain significantly higher titers of antibody against SCG than other strains of mice [16], it seems likely that these mice strains are sensitive to SCG. Thus, DBA/1 and DBA/2 mice would be useful models for future studies of SCG.

Harada et al. further demonstrated that GM-CSF was one of the key factors in reactivity to SCG in DBA/2 mice [15, 17]. Neutralizing GM-CSF using an anti-GM-CSF monoclonal antibody significantly inhibited IFN-*γ*, TNF-*α*, and IL-12p70 elicited by SCG. The splenocytes in various strains of mice showed similar patterns of cytokine production in response to SCG cotreatment in the presence of recombinant murine GM-CSF. The high sensitivity to SGG shown by DBA/1 and DBA/2 mice may be attributable to differences of their regulation of GM-CSF compared with that in other mice.

Harada and Ohno also proposed an interesting model for the mechanism of cytokine induction by SCG in DBA/2 mice [18]. Broadly speaking, SCG directly induces adherent cells to produce TNF-*α* and IL-12p70, whereas cell-cell contact mediated by the association of CD4^+^ T cells expressing LFA-1 and antigen-presenting cells such as dendritic cells expressing ICAM-1 is required for the induction of IFN-*γ* and GM-CSF by SCG.

Neutrophils, macrophages, and dendritic cells express several receptors capable of recognizing beta-glucan in its various forms. Dectin-1, complement receptor 3, lactosylceramide, and scavenger and Toll-like receptors are all candidates that have been reported thus far [19–23]. Among these, dectin-1, which is a C-type lectin, is an archetypical non-Toll-like pattern recognition receptor expressed predominantly by myeloid cells. Dectin-1 can induce its own intracellular signaling and can mediate a variety of cellular responses, such as cytokine production [24].

The magnitude of cytokine induction from bone-marrow-derived dendritic cells (BMDCs) by SCG and the expression level of dectin-1 on BMDCs in DBA/2 mice are both higher than that of other strains of mice. Furthermore, blocking dectin-1 significantly inhibits the induction of TNF-*α* production by SCG. These results suggest that the BMDCs from DBA/2 mice are highly sensitive to SCG-induced cytokine production *in vitro*, and that this sensitivity is related to the expression level of dectin-1 [25].

The molecular mechanism of the enhanced hematopoietic response has been investigated in CY-treated mice (both ICR and C57BL/6 strains) [26]. According to this report, the levels of IFN-*γ*, TNF-*α*, GM-CSF, IL-6, and IL-12p70 were all shown to be significantly increased in SCG-treated splenocytes of CY-treated mice. GM-CSF production in the splenocytes of CY-treated mice was reportedly higher than that in normal mice regardless of SCG stimulation. Neutralizing GM-CSF significantly inhibited the induction of IFN-*γ*, TNF-*α*, and IL-12p70 by SCG. The level of cytokine induction by SCG was modulated by the amount of endogenous GM-CSF produced in response to CY treatment in a dose-dependent manner. The expression of beta-glucan receptors, such as CR3 and dectin-1, was upregulated by CY treatment. Blocking dectin-1 significantly inhibited the induction of TNF-*α* and IL-12p70 production by SCG. Taken together, these results suggest that the key factors in cytokine induction in CY-treated mice are the enhanced levels of both endogenous GM-CSF production and dectin-1 expression. It is very interesting that agents that modulate GM-CSF production and dectin-1 expression, such as CY, can control reactivity to SCG and the expression of various cytokines.

Shibata et al. found that both GM-CSF and TNF-*α* synthesis in DBA/2 mouse splenocytes stimulated with SCG, but not with lipopolysaccharide, were significantly enhanced in the presence of cytochalasin D (CytD), an inhibitor of actin polymerization [27]. On the other hand, Kim et al. pointed out the importance of the role of Toll-like receptor 4 (TLR4) [28]. They examined the effect of SCG on adherent monocytes, such as macrophages and dendritic cells (DCs), and nonadherent lymphocytes, such as T and B cells, and demonstrated that SCG mainly activated DCs and macrophages, but not T and B cells. The role of TLR4 as a membrane receptor of SCG was shown by the impairment of maturation of DCs generated from bone marrow cells of tlr4^−^/^−^ knockout mice and TLR4-mutated C3H/HeJ mice, and by using an anti-MD-2/TLR4 neutralizing antibody. SCG increased the phosphorylation of ERK, p38, and JNK and enhanced nuclear translocation of NF-*κ*B p50/p65 in DCs. These results indicate that SCG activates DCs via MAPK and NF-*κ*B signaling pathways, which are signaling molecules downstream of TLR4.

### 2.2. Low-Molecular-Weight Compounds

**Figure 3:**
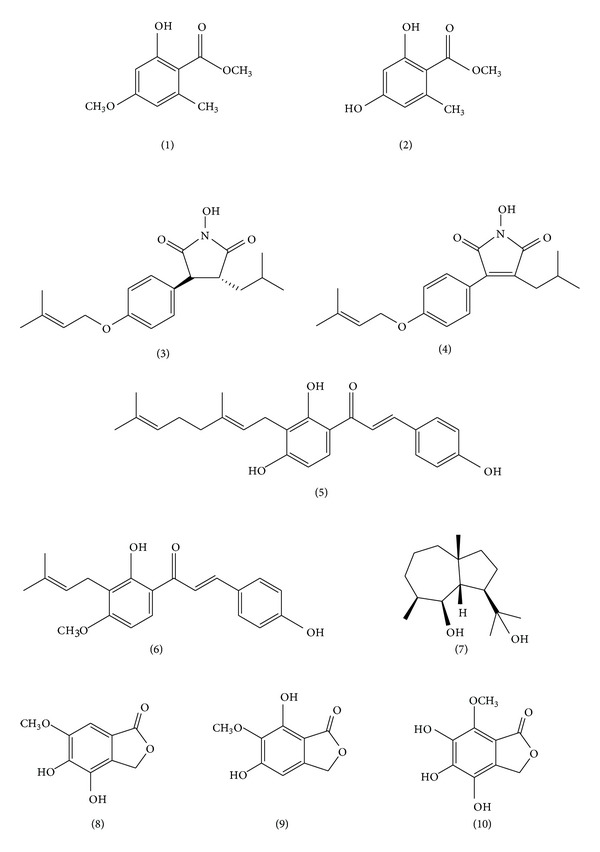
Chemical structure of the low-molecular-weight compounds found in* S. crispa*[29–34].

*S. crispa* possesses a wide range of bioactive metabolites which are products of secondary metabolism (Figure 3).

#### 2.2.1. Antimicrobial Compounds

It has been reported that *S. crispa* produces antibiotic substances. For example, suppression of *Bacillus subtilis* growth on agar media is known to be due to sparassol (methyl-2-hydroxy-4-methoxy-6-methylbenzoate) (1) [29]. Woodward et al. reported that *S. crispa* produced 3 antifungal compounds when submerged in culture in a 2% malt broth. The compounds included sparassol, and two other antifungal compounds, methyl-2,4-dihydroxy-6-methylbenzoate (2) and methyl-dihydroxy-methoxy-methylbenzoate (the positions of substituents were unclear), both of which showed higher antifungal activity than sparassol against *Cladosporium cucumerinum* [30].

A novel compound (4) and a previously known one (3) were isolated from *S. crispa* [31]. Both compounds were shown to inhibit both melanin synthesis and methicillin-resistant *Staphylococcus aureus* (MRSA) growth. The minimum inhibitory concentration (MIC) values of compounds 3 and 4 in the anti-MRSA assay were 0.5 and 1.0 mM, respectively. IC_50_ values of compounds (3) and (4) in the melanin production inhibition assay were 33 *μ*M and 12 *μ*M, respectively. Since the IC_50_ value of an existing whitening agent, arbutin, is reported to be 1.32 mM, these compounds have potential as constituents of cosmetic products.

In the course of screening for compounds that inhibit MRSA growth, Kodani et al. discovered 2 known chalcones, xanthoangelol (5) and 4-hydroxyderricin (6), in the extract of *S. crispa*, which have been previously isolated from the plant *Angelica keiskei*. These compounds showed anti-MRSA activity, and their MICs were 2 and 0.25 mM, respectively. This was the first report of the isolation of chalcones from a representative of the Fungi kingdom [32].

#### 2.2.2. Other Bioactive Compounds

A new sesquiterpenoid was also isolated from *S. crispa *[33]. Its structure was determined to be (3R*, 3aS*, 4S*, 8aR*)-3-(1′-hydroxy-1′-methylethyl)-5,8a-dimethyldecahydroazulen-4-ol (7) by a combination of NMR and ESI-MS analyses. This was the first isolation of an isodaucane-type sesquiterpenoid from a fungus, including mushrooms.

Yoshikawa et al. isolated three novel phthalides, designated hanabiratakelide A (8), B (9), and C (10) in addition to three known phthalides, from the *S. crispa* fruiting body [34]. The 6 isolated compounds were tested for their antioxidant activity. The *in vitro* superoxide dismutase-like activity of the three hanabiratakelides was stronger than that of vitamin C. These compounds also inhibited lipopolysaccharide-stimulated nitric oxide and prostaglandin E2 production by a murine macrophage cell line, RAW264. In addition, the growth of the colon cancer cell lines Caco-2 and colon-26 was significantly inhibited by treatment with all 3 of the hanabiratakelides.

## 3. Pharmacological Aspects of *S. crispa*

### 3.1. Antiviral Activity

Reverse transcriptase (RT) is one of the key enzymes in human immunodeficiency virus (HIV) replication. HIV replication is interfered with when the enzyme is inhibited. Thus, RT inhibitors can be used to treat AIDS. Hot water extracts from the fruiting bodies of 16 species of mushroom, including *S. crispa*, were screened for HIV-1 RT inhibitory activity. The extract of *S. crispa* elicited 70.3% inhibition when tested at a concentration of 1 mg/mL. However, the active component remains unclear [35]. 

### 3.2. Antihypertensive Effects

One of the main causes of stroke is hypertension. Therefore, it is important to avoid high blood pressure as a preventative measure. Yoshitomi et al. investigated not only the preventive effects of *S. crispa* against stroke and hypertension in stroke-prone spontaneously hypertensive rats (SHRSP), but also the mechanism involved by studying the cerebral cortex [36]. SHRSP rats given feed containing 1.5% *S. crispa* had a delayed incidence of stroke and death, significantly decreased blood pressure, and increased blood flow. Moreover, the urinary nitrate/nitrite excretion and the nitrate/nitrite concentration in cerebral tissue were higher than those of control SHRSP rats. In the cerebral cortex, phosphor-eNOS (Ser1177) and phosphor-Akt (Ser473) in *S. crispa*-treated SHRSP rats were increased compared with those of control SHRSP rats. In conclusion, *S. crispa* can ameliorate cerebrovascular endothelial dysfunction by promoting recovery of Akt-dependent eNOS phosphorylation and increasing nitric oxide (NO) production in the cerebral cortex.

In addition, Lee et al. indicated that SCG was able to stimulate NO production as well as enhance the expression of inducible NO synthase (iNOS) from macrophage-like RAW264.7 cells [37]. Since NO production is strongly suppressed by mitogen-activated protein kinase (MAPK) inhibitors, it is likely that SGG-induced NO release is mediated by MAPK.

### 3.3. Antidiabetic Activity

It has been shown that dietary *S. crispa* improves the symptoms of both type 1 and type 2 diabetes. The consumption of a diet containing more than 0.5% *S. crispa* results in significant improvement in diabetes symptoms (body weight loss and increased blood glucose) in ICR mice with STZ-induced diabetes [38]. Furthermore, Yamamoto and Kimura examined the effect of dietary *S. crispa* on KK-Ay mice, an animal model of type 2 diabetes mellitus [39]. The group that was fed 5.0% *S. crispa* diet showed not only a significant decrease of blood glucose and insulin levels, but also a pronounced increase in plasma levels of adiponectin in comparison with a control group. Although the *S. crispa* diet had no effect on body and adipose tissue weights in KK-Ay mice, the size of the mesenteric adipose cells of mice in the *S. crispa* group tended to be smaller than the control group. Thus, the *S. crispa* diet may decrease the adipose cell size in order to increase plasma adiponectin levels. Considering the physiological significance of adiponectin, these findings imply that dietary *S. crispa* has the potential to ameliorate type 2 diabetes.

GPR40 is one of the G protein-coupled receptors, which has 7 transmembrane spanning helical bundles. GPR40 distributes in pancreas and central nervous system. It can be bound by medium- and long-chain fatty acid and activate the intracellular signal pathways, which in turn regulates the function of cells. In pancreatic beta-cell, intracellular calcium concentration elevates when GPR40 is binding to fatty acid, thereby promoting the release of insulin [40]. Yoshikawa et al. demonstrated that a couple of unsaturated fatty acids in *S. crispa* were the agonist of GPR40, which might be used for preventing and treating the diabetes [41].

The normal healing process in healthy individuals takes place at an optimal rate, but it is usually delayed, or even completely impaired in patients with diabetes. Thus, the impaired wound healing that occurs in diabetes mellitus is a major clinical problem. It is also generally accepted that wound repair is an immune-mediated physiologic mechanism. Oral administration of 1,000 mg/kg body weight per day of *S. crispa* for 4 weeks was shown to significantly accelerate wound healing in rats with streptozotocin- (STZ-) induced diabetes, which is an insulin-dependent model of diabetes mellitus (type 1) [42]. Furthermore, in *S. crispa*-treated wounds there were significant increase in macrophage and fibroblast migration, collagen regeneration, and epithelialization compared with a control group. Therefore, the use of *S. crispa* may be extended to the clinical setting, and it may effectively promote wound healing in patients with diabetes.

Yamamoto and Kimura investigated whether oral and topical administration of *S. crispa* could restore effective wound healing in ICR mice with STZ-induced diabetes [38]. Mice consuming a diet containing more than 0.5% *S. crispa* showed significantly improved wound healing. Notably, the rate of wound healing in mice fed a diet containing 2.5% *S. crispa* was almost the same as that in mice treated with topical trafermin (basic fibroblast growth factor formulation). Moreover, topically administered SCG significantly promoted wound healing in mice with diabetes, resulting in a wound contraction ratio of 37% after treatment for 9 days, a result that was superior to that of trafermin.

### 3.4. Antitumor and Anticarcinogenic Activity (except for SCG)

Yamamoto et al. investigated the antitumor effects of a low-molecular-weight (below approximately 8 kDa) fraction (FHL) containing no beta-glucan isolated from a hot water extract of *S. crispa *[43]. The oral administration of FHL (30 mg/kg) to tumor- (sarcoma 180) bearing ICR mice was observed to suppress tumor growth. Furthermore, the IFN-*γ* level in the culture supernatant of splenic lymphocytes from FHL-fed tumor-bearing mice was significantly increased compared to a control group. Tumor-induced angiogenesis in the dorsal air sac (DAS) system was also suppressed by FHL administration. These results suggest that the oral administration of FHL induces antitumor activity through the enhancement of the Th1-response in tumor-bearing mice. Additionally, the antiangiogenic activity of FHL may contribute to its antitumor activity.

Yoshikawa et al. investigated the possible preventive effects of *S. crispa* on azoxymethane-induced colon aberrant crypt foci (ACF) in F344/N rats. *S. crispa* feeding dose-dependently suppressed the malignant changes of ACF by 54% (0.3% group), 64% (1.0% group), and 75% (3.0% group). They concluded that the anticancer-related activity may originate from the aforementioned hanabiratakelides [34].

### 3.5. Antiallergic Activity

Allergic inflammatory diseases, such as food allergy, asthma, hay fever, and atopic dermatitis, are increasing worldwide. Some recent reports have demonstrated antiallergic activities of *S. crispa*. Atopic dermatitis (AD) is a common inflammatory skin disease for which few effective treatments are available. Oral administration of 250 mg/kg body weight per day of *S. crispa* decreased both blood immunoglobulin E (IgE) level and scratching index in Nc/Nga mice with dermatitis induced by a continuous application of 2,4,6-trinitrochlorobenzene [2]. In addition, the antirhinitis properties of *S. crispa* were also investigated in mice [44]. To determine the immunomodulatory activity of oral *S. crispa*, splenocytes obtained from ovalbumin-sensitized BALB/c mice fed 0.25% *S. crispa* were restimulated *in vitro* with the same antigen. The oral *S. crispa* induced IFN-*γ* secretion, but inhibited IL-4 and IL-5 secretion, and suppressed ovalbumin-specific IgE secretion by the splenocytes. The effects of *S. crispa* were further investigated by using an allergic rhinitis model in BALB/c mice. Nasal symptoms, sneezing, and nasal rubbing induced by ovalbumin challenges were inhibited by oral administration of *S. crispa *(36 or 120 mg/kg) in a dose-dependent manner. Furthermore, ovalbumin-specific serum IgE levels were diminished by *S. crispa* treatment in this model. These results demonstrate that *S. crispa *may be effective in suppressing symptoms of allergic rhinitis through suppression of the Th2-type immune response.

Kim et al. reported the effect of a water extract of *S. crispa* (WESC) on mast-cell-mediated allergic inflammation and the possible mechanisms of action using *in vivo* and *in vitro* models [45]. WESC inhibited compound 48/80-induced systemic anaphylaxis and serum histamine release in mice. WESC decreased IgE-mediated passive cutaneous anaphylaxis. Additionally, WESC reduced histamine release and intracellular calcium in human mast cells activated by both phorbol 12-myristate 13-acetate (PMA) and calcium ionophore A23187. Since intracellular calcium plays an important role in the release of histamine and the expression of cytokines, the decreased intracellular calcium levels may be involved in the inhibitory effect of WESC on histamine release. WESC decreased PMA and A23187-stimulated expression of proinflammatory cytokines, such as TNF-*α*, interleukin- (IL-6), and IL-1*β*. The inhibitory effect of WESC on proinflammatory cytokines was shown to be dependent on nuclear factor-*κ*B, extracellular signal-regulated kinase, and p38 mitogen-activated protein kinase. Since the beta-glucan content in WESC was measured to be 39.3%, beta-glucan may be responsible for its antiallergic effects.

## 4. Human Clinical Evaluation

In a study where healthy men were given *S. crispa* powder orally at 300 mg per day for 8 weeks, NK cell cytotoxicity was significantly enhanced without increasing the number of NK cells when compared to preadministration [2]. In addition, Kimura investigated whether dietary *S. crispa* influenced human skin condition [46]. Oral administration of *S. crispa* powder (320 mg/day) for 28 consecutive days dramatically reduced transepidermal water loss, an indicator of the skin barrier condition, while that of a placebo group was unchanged during the testing period. These observations imply that oral administration of *S. crispa* has a positive effect on the skin barrier.

A clinical trial of *S. crispa* used an orally delivered powder (300 mg/day) in patients with several different types of cancer (lung, stomach, colon, breast, ovarian, uterine, prostate, pancreas, and liver cancers) after the patients had received a single course of lymphocyte transfer immunotherapy [47]. Patient assessment of 14 cases after a several month follow-up period (mean: 15 months) revealed that the performance status in 9 cases showed improvement in quality of life, and so forth.

## 5. Conclusions and Future Prospects

*S. crispa* has been described in the literature as a mushroom with great potential for therapeutic applications. The medicinal value of this mushroom is mainly attributable to its abundant 6-branched 1,3-beta-glucan (SCG). By chemical analysis, we found that the primary structure of a purified beta-glucan obtained from liquid cultured mycelium of *S. crispa* was a 6-branched 1,3-beta-glucan, having one branch approximately every 6 residues, with a degree of branching that is relatively less than that of SCG. The effect on tumor- (sarcoma 180) bearing ICR mice was much weaker than that of SCG given by oral administration (data not shown). Furthermore, using 1D- and 2D-NMR spectroscopy, Tada et al. elucidated the fine primary structure of SCG and compared it with sonifilan (SPG) from *Schizophyllum commune*, which is a 6-branched 1,3-beta-glucan and has been used clinically for cancer therapy in Japan, examining differences in the biological effects between these beta-glucans [9]. Though both major structural units are the same beta-(1–3)-glucan backbone with single beta-(1–6)-glucosyl side branching units every 3 residues, the production of IL-6 and TNF-alpha from BMDCs was significantly increased by SCG, whereas these effects were not observed with SPG treatment. These findings may indicate that the biological activities of beta-glucan are attributable not only to its primary structure but also to its conformation.

Though it has been suggested that dietary *S. crispa* is useful for cancer immunotherapy in combination with lymphocyte transplantation [47], the study described in this previous report did not include a randomized control group. Further clinical trials are needed to confirm the pharmacological activity of dietary *S. crispa*. There is still little scientific evidence to explain the differences in responsiveness to beta-glucan in humans. The studies of differences in reactivity to SCG in different animal strains [14, 15, 17] are important from this viewpoint. Furthermore, it is interesting that agents such as CY can control reactivity to SCG, as well as the expression of various cytokines [26]. Further research on reactivity to SCG could provide clues for developing more effective cancer immunotherapies using SCG.

As mentioned above, dectin-1 and TLR4 have been proposed as SCG receptors. It is noteworthy that either treatment with a blocking antibody against dectin-1 [25, 26] or genetic deletion of TLR4 [28] completely prevents SCG-induced DC maturation. These observations might indicate that one signaling pathway did not compensate for the other in SCG-treated DCs, suggesting that both receptors are required for SCG action. However, further analysis of the role of these receptor candidates, which contain complement receptor 3, lactosylceramide, and scavenger-like receptors, in response to SCG would be needed in order to clarify the details of its mechanism of action in DCs [48].

The question has been raised as to how orally administered beta-glucan exerts its effects. The evidence presented in this review clearly indicates that dietary SCG has immunomodulatory actions. Therefore, it must be assumed that orally ingested SCG interacts with either intestinal epithelial cells and/or intestinal DCs, ultimately resulting in the priming or activation of other immune cells.

The antitumor mechanisms of SCG, except for its immunomodulatory actions, have not been well studied. Hence, we tried to elucidate the possible mechanisms of its antiangiogenic effects. As a result, it was demonstrated that SCG has both antiangiogenic functions and antimetastatic effects on neoplasm using different animal models [8]. The antitumor effects of SCG may be partially attributable to its antiangiogenic actions. Numerous reports concerning the antitumor activity of edible mushrooms have taken particular notice of beta-glucan. However, few studies have focused on antitumor components other than beta-glucan. It is worth mentioning that *S. crispa* has been shown to produce some low-molecular-weight constituents with antitumor activity, such as hanabiratakelide [34] and FHL [43].

Recently, Park et al. reported a novel process for nanoparticle extraction of beta-D-glucan from *S. crispa* using insoluble tungsten carbide [49]. This nanoknife method results in high yields of SCG (70.2%) with an average particle size of 150 and 390 nm. The extracted SCG showed a remarkably high water solubility of 90% at room temperature. This nanoknife method could be a potent technology to produce SCG for food, cosmetics, and pharmaceutical industries.

The extract of *S. crispa* might be applied to produce health products such as food, beverage, and antineoplastic drug. Actually, *S. crispa* extractions, resveratrol, and collagen peptide were claimed as antiaging agents and food supplements [50]. Formulation examples of granules and health drinks were disclosed.

Many people in Japan consume *S. crispa*, and to date, no reports of adverse events due to *S. crispa* consumption have been reported. Therefore, dietary treatment with *S. crispa* may prove to be a safe therapy for cancer and other chronic diseases.

## Figures and Tables

**Table 1:** Approximate composition of *Sparassis crispa* (per 100 g dry sample).

Components	Amount (g)
Protein	13.4
Fat	2.0
Ash	1.8
Carbohydrate	21.5
Dietary fiber (DF)	61.2
Beta-glucan from DF	43.5
